# A Four-Year Report on Renal Outcomes Following the Elective Withdrawal of Long-Term Renin-Angiotensin-Aldosterone Blockade in a Cohort of Patients With Otherwise Inexplicable New-Onset and Progressive Acute Kidney Injury

**DOI:** 10.7759/cureus.30794

**Published:** 2022-10-28

**Authors:** Macaulay A Onuigbo

**Affiliations:** 1 Medicine, The Robert Larner, M.D. College of Medicine, University of Vermont, Burlington, USA

**Keywords:** acute kidney injury, late-onset renal failure from angiotensin blockade, raas inhibitors, commitment bias, chronic kidney disease (ckd)

## Abstract

Background

Renin-angiotensin-aldosterone (RAAS) blockade is acclaimed, by consensus, to be renoprotective in both diabetic and non-diabetic chronic kidney disease (CKD). Contradictory reports exist regarding renal and cardiovascular outcomes after stopping RAAS blockade in advanced CKD. A few prospective, non-randomized, cohort studies have demonstrated improvement in kidney function after discontinuation of RAAS blockade. In this study, we investigated renal and mortality outcomes following the elective withdrawal of RAAS blockade after otherwise inexplicable acute kidney injury (AKI).

Methodology

We conducted a retrospective cohort analysis of patients enrolled between February 2018 and May 2021. Kidney function was monitored after elective withdrawal of long-term RAAS blockade in CKD patients presenting with new-onset otherwise inexplicable progressive AKI, defined by a >25% increase in baseline serum creatinine.

Results

In total, 71 patients, 69 Caucasians, one African American, and one Hispanic, were included in the study, with a male-to-female ratio of 42:29, and a mean age of 69.4 (37-95) years. Through February 2022, 12 patients had died, with eight remaining on hemodialysis for end-stage renal disease. Of the remaining 51 patients followed for 706 (40-1,478) days, baseline serum creatinine was 1.30 ± 0.42 (0.66-2.70) mg/dL, peak enrollment serum creatinine was 2.17 ± 1.06 (1.1-8.3) mg/dL (n = 51, p < 0.0001, t = 6.4872, df = 135), and serum creatinine after four years was 1.58 ± 0.54 (0.84-3.3) mg/dL (n = 50, p < 0.0001, t = 5.1805, df = 119). Death in 11 of 12 (91%) patients was from non-renal causes, and most deaths occurred despite improved kidney function.

Conclusions

Our results demonstrate clearly improved renal outcomes in most patients following the elective withdrawal of long-term RAAS blockade in CKD patients with new-onset progressive yet otherwise inexplicable AKI without increased cardiovascular mortality.

## Introduction

Renoprotection from renin-angiotensin-aldosterone (RAAS) blockade in diabetic and non-diabetic chronic kidney disease (CKD) is a well-established and generally accepted cardinal cornerstone of modern medical practice, and this consensus is based on a preponderance of large multicenter randomized clinical trials [1-5]. However, to the contrary, there remain considerable contradicting reports regarding cardiorenal and mortality outcomes following the withdrawal of RAAS blockade, especially in patients with advanced CKD [6,7]. The import of stopping RAAS inhibition versus continuing RAAS inhibition in 10,254 prevalent RAAS inhibitor users (36% females, with a median age of 72 years) who had presented with new-onset estimated glomerular filtration rate (eGFR) <30 mL/minute/1.73 m^2^ body surface area (BSA) was investigated in a Swedish Renal Registry analysis [6]. Median eGFR was 23 mL/minute/1.73 m^2^ BSA [6]. A higher absolute five-year risk of death (40.9% versus 54.5%) and major adverse cardiovascular events (47.6% versus 59.5%) but a reduced risk of kidney replacement therapy (36.1% versus 27.9%) occurred in the group that stopped RAAS inhibitor use [6]. On the other hand, a retrospective cohort analysis in Canada examined the renal, cardiovascular, and mortality outcomes in 49,571 older adults who presented with hyperkalemia (potassium ≥5.3 mEq/L) while on a RAAS inhibitor [7]. The mean age of this Canadian cohort was 79 years [7]. RAAS inhibitor stoppage led to the lowest risk of recurrent hyperkalemia, and without increased short-term incidence of cardiovascular events or all-cause mortality when compared to no intervention [7]. We revisited the conflicting results of these studies in a commentary in the American Journal of Medicine. In this commentary, we reexamined the continuing debate and controversies surrounding renal and cardiovascular outcomes following the preemptive withdrawal of RAAS blockade in patients with advanced CKD [8]. Moreover, a Korean study, reported in 2017, investigated renal death, all-cause mortality, hospitalization for hyperkalemia, and interactive factors as composite outcomes in 2,076 pre-dialysis patients with advanced CKD on RAAS blockers [9]. These outcomes in the patients on RAAS blockers were compared with paired patients not on RAAS blockers using the statistical method of inverse probability of treatment-weighted and propensity score matching [9]. The pre-dialysis patients who continued RAAS blockade displayed higher renal death in propensity score-matched analysis (hazard ratio (HR) = 1.381; 95% confidence interval (CI) = 1.071-1.781; p = 0.013). This outcome was again consistent with the results of the inverse probability of treatment-weighted analysis (HR = 1.298; 95% CI = 1.123-1.500; p < 0.001) [9]. RAAS blockade users had higher composite outcomes in inverse probability of treatment-weighted score (HR = 1.154; 95% CI = 1.016-1.310; p = 0.027), with marginal significance in propensity score-matched analysis (HR = 1.243; 95% CI = 0.996-1.550; p = 0.054) [9]. This Korean study determined that continued RAAS blockade in pre-dialysis patients with advanced CKD may worsen renal outcomes without improving all-cause mortality [9]. The investigators called for further studies regarding withdrawal versus continuing RAAS blockade in these patients [9]. Moreover, the ongoing European STOP-ACEI Trial, a randomized controlled trial comparing cardiorenal outcomes in continuing versus discontinuing RAAS blockade in advanced CKD, may help resolve these vexed questions [10,11].

Nonetheless, the potential nephrotoxicity of RAAS blockade in association with specific clinical scenarios is well described [12-20]. The previously described traditional risk factors for the precipitation of acute kidney injury (AKI), while on concurrent RAAS blockade, include volume depletion, hypotension, renal artery stenosis, concurrent non-steroidal anti-inflammatory drugs (NSAIDs), and heart failure exacerbation [12-20]. Nevertheless, there remains ongoing controversy and debate regarding the role of RAAS blockade in the precipitation of reversible and irreversible AKI in the absence of these traditional risk factors. This author, in 2005, for the first time, described the syndrome of late-onset renal failure from angiotensin blockade (LORFFAB) at the Mayo Clinic Health System in the US Midwest [21]. This syndrome of LORFFAB represented potentially reversible new-onset AKI in CKD patients on concurrent RAAS blockade but in the absence of the previously described traditional risk factors [21]. Since then, collaborating prospective cohort studies have been reported from the same center in the US Midwest (2008), the United Kingdom (2010), and, most recently, Vermont, USA (2021, 2022) to support the existence of LORFFAB [22-25].

In 2021-2022, we reported the results of 40-month renal outcomes following the elective withdrawal of long-term RAAS blockade in 71 patients with otherwise inexplicable new-onset progressive AKI [24,25].

This article was previously posted to Research Square preprint on August 5, 2022.

## Materials and methods

It must be acknowledged that the methods applied in this work are similar to the previous work by the author dating back from our first publication in the journal Medical Science Monitor, published in 2005, to the most recent report in the journal Medical Research Archives, published in 2021 [21,22,24,25].

In 2021, we reported the results of 40-month renal outcomes following the elective withdrawal of long-term RAAS blockade in 71 patients (69 Caucasians, one African American, and one Hispanic) with a male-to-female ratio of 42:29, and a mean age 69.4 (37- 95) years who had presented with new-onset, progressive yet otherwise inexplicable AKI [24,25]. We have since continued to follow up on kidney function, cardiovascular, and all-cause mortality outcomes in this patient cohort. These CKD patients were on a stable dose of RAAS blockade for >90 days and had presented with new-onset progressive >25% increase in baseline serum creatinine in the absence of the traditional risk factors described above. Baseline serum creatinine was defined as the last available for each patient before presentation with increasing serum creatinine, usually within three months of this presentation [21,22]. Kidney function, as measured by serum creatinine, was rechecked one to four weeks after drug discontinuation, and then every two to three months. eGFR was derived using the CKD-EPI equation and was concurrently reported in the electronic medical record. Patients were excluded if they had an increase in the dose of RAAS blockade in the preceding three months, or had evidence of hypotension or orthostatism, perioperative patients, volume depletion, heart failure exacerbation, concurrent exposure to NSAIDs, overt infections, other acute medical conditions, and/or if there was a demonstrated presence of hydronephrosis on renal sonogram wherever available [21,22]. The patients’ primary care providers collaboratively followed up with the patients after drug discontinuation with close monitoring of blood pressure. Anti-hypertensive therapy was adjusted as indicated with the substitution of “kidney-friendly” agents such as calcium channel blockers, beta-blockers, alpha-blockers, and vasodilators such as hydralazine and minoxidil [21,22].

At the end of February 2022, we completed a 48-month review of renal, cardiovascular, and all-cause mortality outcomes in this patient cohort.

Statistical analysis

Concerning quantitative analysis, for all continuous variables, the results are reported as means ± standard deviation (SD), and the ranges of data are simultaneously displayed in parenthesis. The differences between means were estimated using the Student’s t-test. A p-value of <0.05 was considered statistically significant. Furthermore, for differences within groups, the two-tailed Student’s t-test was utilized in the statistical analysis. Conversely, for differences between groups, we used the unpaired Student’s t-test. Other study results are presented as figures, tables, and straight-line graphs. Translational changes in mean serum creatinine values of the cohort at baseline, at study entry, and after four years of follow-up are represented as straight-line graphs. Additionally, the serum creatinine trajectory of a particular patient was depicted as straight-line graphs. Finally, the trajectory of eGFR by the CKD-EPI equation over time, of another patient, is displayed as a straight-line graph.

## Results

The original Vermont cohort consisted of 71 patients (69 Caucasians, one African American, and one Hispanic) with a male-to-female ratio of 42:29, and a mean age of 69.4 (37-95) years at enrollment [24,25]. Medical comorbidities included diabetes mellitus (37) and hypertension (66). At study entry, the patients were mostly asymptomatic. The mean duration of RAAS blockade before drug discontinuation was 2,057 (112-4,043) days. The mean duration of follow-up of this cohort, following drug discontinuation, as of May 2021, when the first analysis was completed, was 580 (17-1,245) days [24,25].

Renal outcomes after discontinuation of RAAS blockade after four years

**Figure 1:**
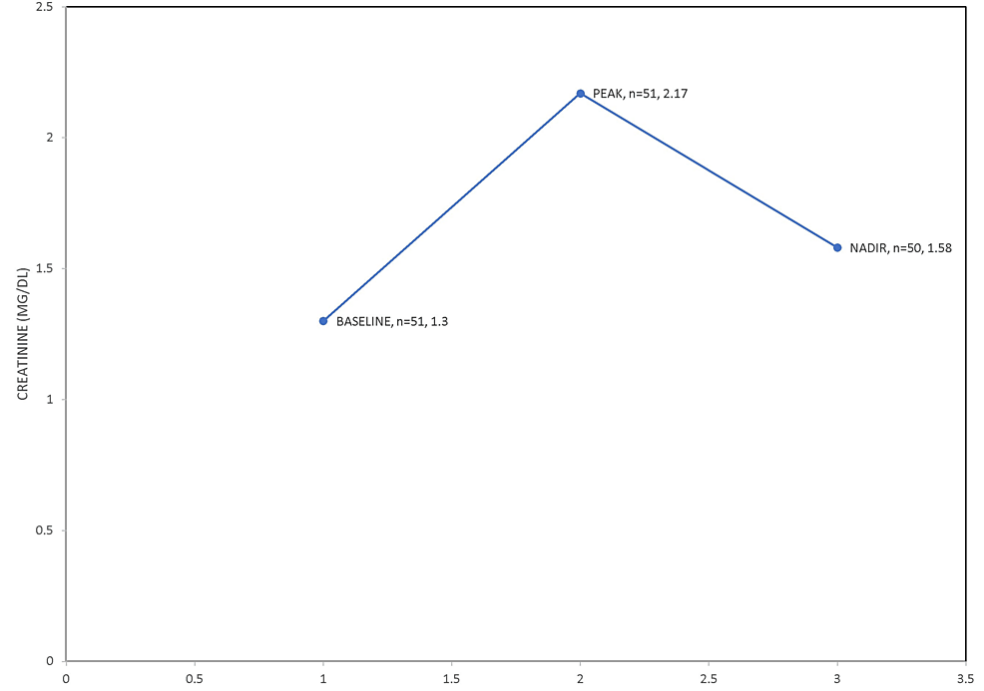
Results of four-year cohort analysis of mean serum creatinine trajectory.

At the end of February 2022, 48 months from the start of this study, after excluding 12 patients in the cohort who had died, and eight patients who had since started maintenance hemodialysis for end-stage renal disease (ESRD), the remaining 51 patients were followed up for a mean of 706 (40-1,478) days. Of this subgroup of 51 patients, baseline serum creatinine was 1.30 ± 0.42 (0.66-2.70) mg/dL (n = 51), peak serum creatinine at entry into the cohort study with new-onset progressive AKI was 2.17 ± 1.06 (1.1-8.3) mg/dL (n = 51, p < 0.0001, t = 6.4872, df = 135), and the latest serum creatinine available at the end of 48 months was 1.58 ± 0.54 (0.84-3.3) mg/dL (n = 50, p < 0.0001, t = 5.1805, df = 119 (Figure 1).

Mortality outcomes following discontinuation of RAAS blockade after four years

There were 12 (17%) deaths. The causes of death are listed in Table 1. The causes of death in 11 of 12 (92%) patients, apart from one patient who decided not to start hemodialysis for ESRD, were from non-renal causes (Table 1). Furthermore, most deaths occurred despite improved kidney function following the discontinuation of concurrent RAAS blockade (Table 1). The levels of serum creatinine at the time of death, as shown in Table 1, ranged from 0.70 mg/dL to 4.91 mg/dL. Serum creatinine was less than 2.0 mg/dL in six of 12 (50%) patients who died (Table 1).

**Table 1:** Causes of death with serum creatinine at the time of death (n = 12). PEA: pulseless electrical activity; NSTEMI: non-ST-elevation myocardial infarction; VT: ventricular tachycardia; CHF: chronic heart failure; GI: gastrointestinal

Cause of death	Number	Serum creatinine (mg/dL) at death
Undifferentiated pharyngeal squamous cell carcinoma	1	1.0
Decision not to start hemodialysis	1	4.91
Cardiac arrest (PEA)	1	1.48
NSTEMI + VT arrest	1	1.64
CHF exacerbation	2	4.73; 4.18
Closed-loop bowel obstruction	1	1.2
GI bleed + hemorrhagic shock	1	2.17
GI bleed	1	4.03
Metastatic renal cell carcinoma	1	0.70
Non-ischemic cardiomyopathy + atrial fibrillation	1	1.99
Aspiration pneumonia + failure to thrive	1	2.33

A case summary of kidney function recovery following discontinuation of losartan 100 mg daily

**Figure 2:**
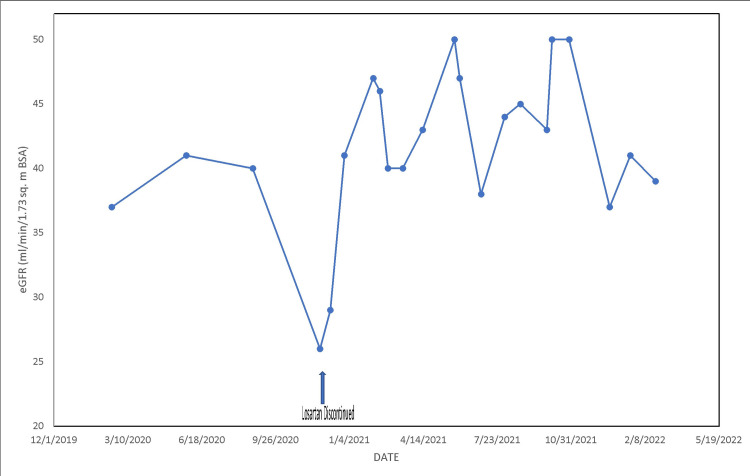
Trajectory of estimated glomerular filtration rate in a female chronic kidney disease patient from February 2020 to February 2022 following elective discontinuation of five years of losartan 100 mg daily in December 2020 (arrow).

In November 2020, a 73-year-old female patient with an extensive medical and surgical history that included hypertension, type II diabetes mellitus with microalbuminuria, previous coronary artery bypass, previous pacemaker placement with atrial fibrillation, lower extremity edema, sleep apnea, CKD III, aortic valve disease s/p aortic valve replacement with a Bjork Shiley valve in 1984, complicated by methicillin-resistant *Staphylococcus aureus* endocarditis, and s/p replacement of the Bjork Shiley prosthesis with a bioprosthetic valve in 2006 had presented to the Nephrology Clinic with reduced appetite and reduced urine output without other systemic symptoms. She had previously been on lisinopril for hypertension, which was discontinued in 2015 after she developed a cough. She was then switched to losartan, 100 mg daily, about five years prior to the presentation. Serum creatinine, which was 1.30 mg/dL in May 2020 and 1.31 mg/dL in August 2020, had increased to 1.87 mg/dL by November 2020. She was not dehydrated, not on NSAIDs, was normotensive, and was not orthostatic, with a blood pressure of 140/70 mmHg. Urinalysis was normal. Workup for AKI was negative for urine protein electrophoresis and serum protein electrophoresis. Anti-nuclear antibody was negative, and C3 and C4 complements were normal. Following this negative AKI workup for inexplicable AKI on CKD, losartan 100 mg daily was discontinued early in December 2020. The plan was to consider increasing the dose of metoprolol and clonidine by the primary care provider, as indicated by subsequent blood pressure monitoring. Kidney function promptly improved, appetite returned to normal, and serum creatinine remained in the 1.2-1.40 mg/dL range from late December 2020 through to February 2022 (Figure 2). The urine albumin creatinine ratio was 0.05 mg/mg in June 2020 while on losartan; it was 0.05 mg/mg in July 2021, seven months after the elective withdrawal of losartan. The patient presently remains an active 74-year-old female in February 2022, with controlled hypertension on metoprolol 100 mg twice daily. Moreover, in January 2021, she resumed metformin 1,000 mg twice daily for type II diabetes mellitus with improved kidney function. Metformin was withdrawn in November 2020 following progressive AKI.

**Figure 3:**
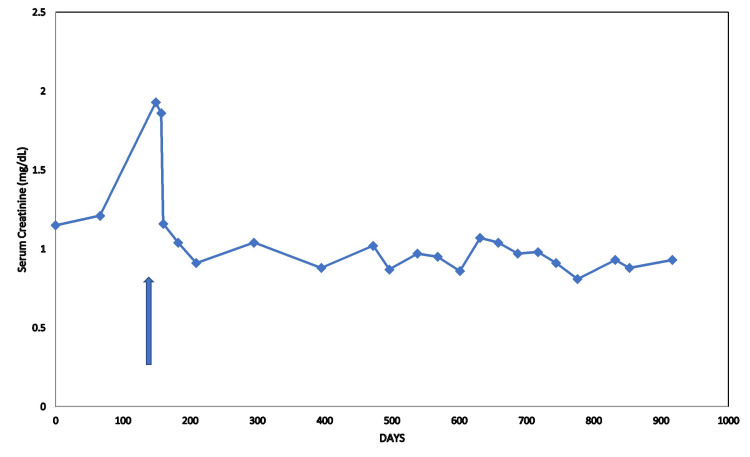
Serum creatinine trajectory in a female chronic kidney disease patient after the discontinuation of years of olmesartan 40 mg daily in December 2020 (arrow) followed through to February 2022.

Another female patient, with a medical history of hypertension and CKD, had similarly exhibited new-onset otherwise inexplicable AKI, confirmed on repeat laboratory testing. The patient had been on olmesartan 40 mg daily for years. Olmesartan was preemptively discontinued in January 2020 and creatinine quickly improved and had since remained stable through February 2022 (Figure 3).

## Discussion

We have, by this report, reaffirmed our previous prospective, non-randomized, cohort observations that in patients on long-term RAAS blockade who present with new-onset progressive and otherwise inexplicable AKI, the elective withdrawal of RAAS blockade is appropriate and results in significant renal salvage without any worsened cardiovascular or all-cause mortality [21,22,24,25]. Once again, we must restate our previous narrative that our observations relate to CKD patients who present with new-onset and progressive AKI on CKD at the time the RAAS blockade was discontinued. This is very different from the ongoing STOP-ACEI Trial where in a randomized study, RAAS blockade is withdrawn versus continued in patients with advanced CKD but without the specific requirement of presentation with new-onset progressive AKI at the time of enrollment in the trial [10]. Moreover, our observations do not necessarily provide any answers to the questions, debates, and controversies that continue to brew from the results of such studies, such as those reported in the Swedish Renal Registry, the Canadian retrospective cohort study, or the Korean multicenter retrospective cohort study, referred to above, in the introduction [6,7,9].

Raine, in 1990, described anatomical changes in the renal microcirculation after carrying out pathologic examination of kidney biopsies from patients who had experienced AKI while on angiotensin-converting enzyme (ACE) inhibition [26]. He postulated that the presence of microvascular renal arteriolar narrowing, different and distinct from macroscopic renovascular disease as in renal artery stenosis, and therefore not evident on conventional renal angiography, could explain the observed deterioration of renal function in some patients on ACE inhibition [26]. This author subsequently hypothesized in 2008 that the syndrome of LORFFAB, which incidentally was reported in mostly older >65-year-old patients, could be pathogenetically explained on the basis of the existence of progressive microvascular renal stenosis (mRAS) [22]. The presence of such progressive mRAS could have stimulated heightened angiotensin II production from the adrenal glands, with the resulting propensity of progressive AKI with RAAS blockade from the resulting selective loss of efferent arteriolar tone, decreased glomerular filtration rate, and, consequently, rising serum creatinine [22]. Most intriguingly, we recently read with excitement and exaggerated interest the original article by Watanabe et al. in the Journal of Clinical Insights that described a link between inhibition of the renin-angiotensin system and concentric hypertrophy of renal arteries in mice and humans [27]. This report, we must admit, provides the most plausible supporting evidence to our previous hypotheses and expressed opinions on LORFFAB, as well as the concept of microvascular renal artery stenosis [22,27].

To stop or not to stop RAAS blockade in otherwise inexplicable acutely worsening AKI on CKD?

This vexatious question poses a recurring dilemma to nephrologists, cardiologists, and general physicians around the world. This author had been booed at Grand Round presentations by Professors of Medicine when revisiting this topic at a major US medical school several years ago. This author subsequently participated in a well-reported international debate hosted by the American Society of Nephrology at its Annual Congress in San Diego, CA, USA in November 2012, under the chairmanship of Richard J. Glassock MD FASN, Professor, Emeritus Professor of Medicine at the Geffen School of Medicine, at the University of California at Los Angeles, USA, a world-renowned nephrologist. The debate was on continuing versus discontinuing the RAAS blockade in AKI.

Our 2011 critical appraisal of the consensus-driven accumulated evidence base that supports the concept of renoprotection with RAAS blockade did raise very serious questions and reasons for skepticism in wholeheartedly following this mantra [28]. For example, some RAAS blockade randomized controlled trials (RCTs) were of short duration, and some reported studies were indeed as short as only 8-12 weeks [29]. The majority of these RAAS blockade RCTs frequently recruited relatively younger patients usually with well-preserved baseline renal function. Indeed, despite exclusion criteria that suggested higher inclusion serum creatinine cut-offs of >2.5 mg/dL, most reported drug trials actually enrolled subjects with much lower levels of serum creatinine. Consequently, baseline serum creatinine of most of the RAAS blockade renoprotection RCTs was in the 1.3-1.5 mg/dL range [28,30-33]. Such near-normal levels of serum creatinine in the recruited study participants raised questions and concerns about some form of recruitment selection bias toward patients with de facto preserved baseline kidney function, than otherwise, in these drug trials [28,30-33]. Moreover, most of these RAAS blockade reno-protection RCTs recruited middle-aged patients, often with minimal burden of comorbidities, with previous known drug adherence and tolerance to ACE inhibitors or angiotensin II receptor blockers (ARBs) [28,30-33]. Furthermore, some of the RAAS blockade renoprotection RCTs had other design and statistical methodological flaws that include the rampant use of questionably unrelated composite endpoints with surrogate and non-surrogate indices and markers. There is evidence that the use of such surrogate and non-surrogate indices of patient outcomes in combined endpoints raises very strong analytical questions regarding the internal validity of the statistical analyses. These concerns include the looming large probability that such analyses will result in the phenomenon of ecologic fallacy and Simpson’s paradox [31,34-36]. Lastly, some RAAS blockade renoprotection RCTs described very high treatment drug discontinuation rates. Again, in the RENAAL trial, the trial drug, losartan, was discontinued in 46.5% of the study arm versus a 53.5% drug discontinuation rate in the placebo arm [3,28,33]. The impact of such high rates of trial drug discontinuation on the validity of study data analysis and consequent study conclusions is very concerning and can only remain speculative [28,33].

Similarly, in 2013, in a comprehensive review of the RAAS blockade renoprotection RCTs and the evidence-base resulting therefrom, we perceived and speculated that current renoprotection strategies with RAAS blockers alone were not the “magic bullet” nor were they indeed the perfect solutions or the cure-all to the problems of cardiorenal protection [37]. We highlighted the limitations of the RAAS blockade RCTs vis-à-vis renoprotection, as already elaborated in the preceding paragraph. Furthermore, we highlighted the absence of elaborate and systematic assessment methods for the reporting of individual patient-level adverse drug events in the RAAS blockade RCTs [37]. Furthermore, we emphasized the preponderance of the literature that supported the existence of other multiple and independent putative pathogenetic mechanisms that drive both diabetic and non-diabetic CKD progression [37]. Our conclusion, therefore, from the foregoing submissions and observations was that the notion that the blockage of a single molecule, angiotensin II, would represent the cure-all for renoprotection, was most likely untenable and that indeed such a claim represented an overreach [37].

Finally, the recent introduction of new cardio-renoprotective agents including the sodium-glucose cotransporter 2 inhibitors, the glucagon-like peptide-1 agonists, a new non-steroidal mineralocorticoid antagonist, and dipeptidyl peptidase 4 inhibitors, all based on new evidence of credible renoprotection and cardioprotection, following positive outcomes from newly reported RCTs, and with all these agents administered as add-on therapy to angiotensin blockade, is most noteworthy. These RTCs clearly and arguably, lend strong credence to the limitations of ACE inhibitors and ARBs in cardiorenal protection in hypertension, CKD, and heart failure [38-40].

The strengths of this report include the availability of a comprehensive set of very detailed individual patient-level clinical and follow-up real-time laboratory data. Our report is clearly limited by being a retrospective, observational, and single-center experience. Furthermore, our patients were mostly Caucasians, the sample size was small, and, most importantly, this was not a randomized controlled trial.

## Conclusions

Nonetheless, given all the above considerations and exigencies, and cognizant of the physician oath “To Do NO Harm,” we make the following submissions. All physicians agree, without any exceptions, that diabetic patients who presented to the Emergency Department, unconscious and in a hypoglycemic coma, will have insulin promptly withheld, and they would be immediately administered intravenous glucose infusions as a life-saving maneuver. Similarly, all physicians would agree, again without any exceptions, that patients on warfarin for previous life-threatening pulmonary embolism, who presented to the Emergency Department with life-threatening hematemesis will have warfarin promptly withheld, and this is followed instantaneously by the administration of parenteral vitamin K, intravenous fresh frozen plasma, and maybe platelets. On a similar line of argument, we would argue that all physicians must agree that discontinuing RAAS blockade in any CKD patient who presents with acutely worsening and otherwise inexplicable AKI is indeed appropriate. Physicians should not allow commitment bias and confirmation bias to engender them from breaking the physician oath of doing no harm to patients.

AKI exacerbations are known to occur with patients on RAAS inhibition who experience any of the following concurrent conditions: dehydration, volume depletion, infections, renal artery stenosis, use of NSAIDs, and over-diuresis. Additionally, there could be the intrusion of other new simultaneous causes of AKI such as obstructive uropathy or other systemic or renal diseases that have nothing to do with ongoing RAAS blockade. We have argued that self-selected CKD patients on concurrent RAAS blockade who exhibit new-onset acute and progressively worsening AKI must be evaluated and managed differently compared to patients who simply have albeit stable but advanced CKD and are on RAAS blockade. We have argued that unquestionably, in these self-selected patients with new-onset progressive AKI, after excluding the well-known traditional precipitating factors described above, and other renal or systemic conditions, that in all other cases with otherwise inexplicable acute kidney injury, the RAAS blockade must be discontinued.

We would argue, unquestionably, that a large randomized controlled multicenter study on the impact of the elective withdrawal of concurrent RAAS blockade in CKD patients presenting with otherwise inexplicable progressive AKI on renal salvage and on both all-cause and cardiovascular mortality is most urgently warranted. We would be excited to join this research effort and collaboration. Nevertheless, until such a trial is completed, we posit that our four-year report strongly supports the preemptive discontinuation of RAAS blockade in such clinical scenarios. There is evidence from our study for improved renal outcomes without any increase in all-cause and/or cardiovascular mortality. We rest our case!
